# Extracellular Vesicles and Tunnelling Nanotubes as Mediators of Prostate Cancer Intercellular Communication

**DOI:** 10.3390/biom15010023

**Published:** 2024-12-27

**Authors:** Jessica K. Heatlie, Joanna Lazniewska, Courtney R. Moore, Ian R. D. Johnson, Bukuru D. Nturubika, Ruth Williams, Mark P. Ward, John J. O’Leary, Lisa M. Butler, Doug A. Brooks

**Affiliations:** 1 Clinical and Health Sciences, University of South Australia, Adelaide, SA 5000, Australia; 2 Department of Histopathology, Trinity College Dublin, D02 PN40 Dublin, Ireland; 3 South Australian ImmunoGENomics Cancer Institute and Freemasons Centre for Male Health and Wellbeing, University of Adelaide, Adelaide, SA 5005, Australia; 4 Solid Tumour Program, Precision Cancer Medicine Theme, South Australian Health and Medical Research Institute, Adelaide, SA 5000, Australia

**Keywords:** prostate cancer (PCa), tunnelling nanotubes (TNTs), extracellular vesicles (EVs), cellular bridges, androgen receptor (AR), ezrin

## Abstract

Prostate cancer (PCa) pathogenesis relies on intercellular communication, which can involve tunnelling nanotubes (TNTs) and extracellular vesicles (EVs). TNTs and EVs have been reported to transfer critical cargo involved in cellular functions and signalling, prompting us to investigate the extent of organelle and protein transfer in PCa cells and the potential involvement of the androgen receptor. Using live cell imaging microscopy, we observed extensive formation of TNTs and EVs operating between PCa, non-malignant, and immune cells. PCa cells were capable of transferring lysosomes, mitochondria, lipids, and endoplasmic reticulum, as well as syndecan-1, sortilin, Glut1, and Glut4. In mechanistic studies, androgen-sensitive PCa cells exhibited changes in cell morphology when stimulated by R1881 treatment. Overexpression assays of a newly designed androgen receptor (AR) plasmid revealed its novel localization in PCa cellular vesicles, which were also transferred to neighbouring cells. Selected molecular machinery, thought to be involved in intercellular communication, was investigated by knockdown studies and Western blotting/immunofluorescence/scanning electron microscopy (SEM). PCa TNTs and EVs transported proteins and organelles, which may contain specialist signalling, programming, and energy requirements that support cancer growth and progression. This makes these important intercellular communication systems ideal potential targets for therapeutic intervention.

## 1. Introduction

Prostate cancer is the second most common form of cancer in males worldwide, and the incidence of this disease is predicted to double globally by 2040 [1]. Currently, there are over 1.4 million new cases of prostate cancer diagnosed globally each year, and more than 330,000 deaths [2]. The androgen receptor (AR) nuclear transcription factor pathway is pivotal for prostate cancer development and progression, where the binding of testosterone to the AR activates downstream signalling cascades to regulate cancer cell proliferation, survival, and differentiation [3,4,5]. The dependence of prostate cancer on androgen biology and its role in disease progression and metastasis makes targeting AR signalling a cornerstone of prostate cancer therapeutic intervention [6]. However, this is far from curative, and it is important to gain a better understanding of AR biology, its effects on cancer growth/progression and, importantly, how it is involved in critical cancer cell intercellular communication pathways. 

It is becoming increasingly evident that intercellular communication is a critical process supporting prostate cancer cell differentiation and dissemination [7,8,9]. Tunnelling nanotubes (TNTs) and extracellular vesicles (EVs) are emerging as key mediators of intercellular communication, which cancer cells may utilise, respectively, for the direct short-range transfer of constituents/information, or for transfer over longer distances. While TNTs and EVs are well-recognised entities, the molecular mechanisms controlling their biogenesis and cargo transport are less well defined, particularly in prostate cancer cell biology. 

TNTs represent dynamic cellular membrane protrusions (50–200 nm diameter) that can facilitate direct cell-to-cell communication between distant cells (10–200 µm) and be stabilised to form more substantive cellular bridges (1–20 µm diameter) [10]. Within the tumour microenvironment, this may enable the transfer of diverse cargo that includes proteins, metabolic substrates, such as lipids and sugars, genetic material, cytosolic signalling machinery, and whole organelles between neighbouring cancer cells and non-malignant cells [11]. While the molecular mechanisms governing TNT formation are yet to be discovered, evidence is emerging to suggest a role for cytoskeletal rearrangements and molecular interactions in their genesis [12]. Indeed, ezrin and other actin binding proteins have increased expression in prostate cancer and may play a critical role in linking membrane to cytoskeletal microfilaments [13] and TNT formation. TNTs have been postulated to play a role in prostate cancer pathogenesis, including promoting tumour heterogeneity, facilitating the dissemination of oncogenic signals, and mediating therapeutic resistance [14,15]. TNT and the cellular bridge-mediated transfer of organelles and other cell constituents may contribute to cancer progression by energy transfer, aiding immune evasion, and be directly involved in the subversive modification of the tumour microenvironment [14].

EVs may also serve as key mediators of intercellular communication in prostate cancer, facilitating the exchange of molecular cargo and programming over longer distances. As a generic descriptor for exosomes, nano-vesicles, micro-vesicles, and other vesicular carriers that are small, and membrane-bound compartments released from cells, EVs can transport biomolecules, cytosolic constituents, nucleic acids, metabolic substrates, and other organelles [16]. EVs can be derived from either endosomes or the cell surface and can be detected in a range of biological fluids, making them attractive targets for non-invasive cancer diagnosis and monitoring [17,18]. Extracellular vesicle-mediated communication is thought to be important in prostate cancer progression, with EVs being implicated in modulating tumour–stromal interactions and immune functions, facilitating metastatic spread, and conferring resistance to therapy [19,20,21].

Intercellular communication within the prostate cancer microenvironment plays a crucial role in disease progression and therapeutic response. Tunnelling nanotubes, cellular bridges, and EVs represent potential mechanisms by which cells exchange information or resources to influence tumour growth, metastasis, and treatment resistance. Understanding the intricate interplay between these pathways and the androgen receptor signalling cascade is paramount for developing diagnostic strategies and targeted therapies to implement personalised management for patients with prostate cancer. Here, we have investigated the dynamics of TNT/cellular bridge and EV formation in prostate cancer cells, visualised the organelle cargo and exchange of AR between cancer and non-malignant cells, and examined some of the molecular machinery that may be involved in TNT/EV biogenesis.

## 2. Materials and Methods

### 2.1. Cell Culture

Human prostate cell lines PNT1a (#95012614), LNCaP (#89110211), 22RV1 (#05092802), and PC 3 (#90112714) were obtained from the European Collection of Authenticated Cell Cultures (ECACC) via CellBank (NSW, Australia), while PWR-1E (CRL11611), DU145 (HTB-81), and pancreatic BxPC-3 cells (CRL1687) were obtained from the American Type Culture Collection (ATCC) via InVitro Technologies (VIC, Australia). THP-1 monocyte cells were also procured from InVitro Technologies (VIC, Australia). PNT1a, LNCaP, 22RV1, BxPC-3, and THP-1 cells were maintained in RPMI 1640 medium (Gibco^®^, Thermo Fisher Scientific Australia Pty Ltd., VIC, Australia), the PC 3 cell line was maintained in Ham’s F12K medium (Gibco^®^, Thermo Fisher Scientific Australia Pty Ltd., VIC, Australia), and the DU145 cell line was cultured in MEM with 1 mM sodium pyruvate and 2 mM L-glutamine, all supplemented with 10% (*v*/*v*) foetal bovine serum (FBS; Moregate Biotech Pty Ltd., QLD, Australia). PWR-1E cells were cultured in Keratinocyte Serum Free Media (Gibco^®^, Thermo Fisher Scientific Australia Pty Ltd., VIC, Australia). Cell culture media were replenished every three days and cells sub-cultured at ~80% confluency. Cell cultures were maintained at 37 °C with 5% CO_2_. The cell lines were authenticated by short tandem repeat profiling and were tested to be negative for mycoplasma contamination using a MycoAlert Mycoplasma detection kit (Lonza Bioscience, NSW, Australia). For all experiments, the cells were seeded at densities calculated to reach ~70% confluency at the experimental end-point (typically, PNT1a; 5.6 × 10^3^ cells/cm^2^, PWR-1E; 9.2 × 10^4^ cells/cm^2^, LNCaP; 2.8 × 10^4^ cells/cm^2^, 22RV1; 1.75 × 10^4^ cells/cm^2^, PC 3; 1.3 × 10^4^ cells/cm^2^, DU145; 1.2 × 10^4^ cells/cm^2^, THP-1; 1 × 10^5^ cells/cm^2^). THP-1 monocytes were activated and matured to macrophages at the time of seeding by the addition of 40 ng/mL phorbol-12-myristate-13-acetate (PMA; Sigma-Aldrich Pty Ltd., NSW, Australia) in DMSO to the cell culture medium. For co-culture experiments, cells were differentially stained or transfected as per relevant protocol below, mixed in a 1:1 ratio and seeded for 48 h before downstream applications.

### 2.2. Plasmid Construction and DNA Transfection

Cells were transfected with LAMP1-GFP and -RFP DNA plasmids as previously described [22]. LAMP1 plasmids were designed to contain LAMP1 (NCBI Reference Sequence NP_005552.3) with GFP N-terminally tagged. The pcDNA3.1_AR-mCherry plasmid was designed to contain the Homo sapien androgen receptor (AR), transcript variant 1 (NCBI Reference Sequence NM_000044.4), whereby the stop codon was removed and continued with the mCherry fusion tag to preserve both the 3′ and 5′ UTR (Figure S1). The required plasmid transcript was produced, sequenced, and subcloned into the pcDNA3.1 vector using GeneArt (Life Technologies, Regensburg, Germany). AR-mCherry was transfected into cells 24 h post-seeding with approximately 250 ng DNA/cm^2^ surface area using Lipofectamine 2000 (Invitrogen^TM^, Thermo Fisher Scientific Australia Pty Ltd., VIC, Australia) as per the manufacturer’s instructions. CellLight Bacmam 2.0 transfections for Actin-GFP and PM-RFP were conducted via the manufacturer’s instructions (additional information in Table 1).

### 2.3. Fluorescent Labelling

To stain cell plasma membrane (CellMask), mitochondria (Mitotracker), ER (ERtracker), lipids (BODIPY), and lysosomes (Lysotracker), cell culture media were changed to serum-free medium and cells were stained as per the manufacturer’s instructions (additional information in Table 1). For differential DiO/DiD labelling, the cells were trypsinised and washed in PBS, and 5 µL of DiD or DiO wass added per 1 mL of cell suspension. The cells were incubated with dye for 20 min at 37 °C with gentle inversion every 5 min, then centrifuged and washed with warm cell culture medium twice before seeding.

### 2.4. Immunofluorescence

Live cells were fixed with 4% paraformaldehyde (PFA) in PBS containing 4% (*w*/*v*) sucrose for 10 min, then washed and subsequently blocked/permeabilised with 5% (*w*/*v*) bovine serum albumin (BSA) (Sigma-Aldrich Pty Ltd., NSW, Australia) and 0.05% (*w*/*v*) saponin (Sigma-Aldrich Pty Ltd., NSW, Australia) for 1 h at room temperature (RT). The cells were then incubated with primary antibody (see Table 1) in block overnight at 4 °C with gentle agitation, washed and further incubated with AlexaFluor conjugated secondary antibody and Hoechst for 1 h at RT. Coverslips were mounted on the slides using Prolong Glass Antifade (P36980; Thermo Fisher Scientific Australia Pty Ltd., VIC, Australia).

### 2.5. Confocal Microscopy and Live Cell Imaging

Fluorescence microscopy was performed using a Nikon A1+ confocal microscope (Nikon, Tokyo, Japan) equipped with a LU-N4/LU-N4S 4-laser unit (403, 488, 561 and 638 nm), using a Plan Apo λ 60 × oil-immersion objective lens (1.4 N.A.) at 1.2 AU pinhole with NIS Elements software (v4.5, Nikon). Each experiment was repeated three times, with the images of ten cells captured per replicate. Imaging was performed using a resonant scanner at a 512-pixel resolution, piezo z-stage, and 2× line averaging with 3× zoom (0.14 μm/px), and 18 z-steps of 0.4 μm were imaged, with 100 3D frames obtained (~2.5 min per cell). Live cell imaging was captured with either a Galvano scanner at 2048 or 512-pixel resolution, piezo z-stage, 1× zoom (0.15 μm/px), for 1 h and 30 min with no delay.

### 2.6. Androgen Treatment

Cell culture medium was replaced with fresh RPMI supplemented with 10% charcoal stripped FBS (Gibco^®^, Thermo Fisher Scientific Australia Pty Ltd., VIC, Australia) and LNCaP cells cultured for 24 h at 37 °C with 5% CO_2_. 10 nM synthetic dihydrotestosterone (DHT) (R1881; Sigma-Aldrich Pty Ltd., NSW, Australia), or vehicle (0.01% *v*/*v* ethanol (EtOH)), was added to the culture medium, and the cells were incubated with vehicle or R1881 for either 48 h before imaging, or added immediately during live cell imaging capture. 

### 2.7. Scanning Electron Microscopy

DU145 cells were seeded onto glass coverslips, rinsed in PBS, then fixed in 2.5% glutaraldehyde + 4% paraformaldehyde in PBS for 1 h. The cells were then rinsed with PBS and treated with 1% Osmium Tetroxide for 1 h, followed by a rinse with PBS. The cells were then dehydrated in a graded series of EtOH and critical point dried (CPD) using a Tousimis 931 CPD (Tousimis, MD, USA). Coverslips were mounted on stubs using carbon tabs, then platinum coated. Scanning electron microscopy was performed on the XL30 FEG SEM (Phillips, The Netherlands).

### 2.8. Protein Extraction and Quantification

Cells were washed with ice cold PBS (ThermoFisher Scientific; catalogue #10010023), then scraped in 200 µL of RIPA Buffer (ThermoFisher Scientific; catalogue #89901) with an inhibitor cocktail (HALT™, ThermoFisher Scientific; catalogue #78440). The cells were syringed with a 26 G needle 3–4 times, and the cell lysate was centrifuged at 10,000× *g* for 10 min at 4 °C. The supernatant was kept at −30 °C until required. The protein concentration of the lysates was quantified using a Pierce^TM^ BCA Protein Assay Kit (ThermoFisher Scientific, catalogue #23225) as per the manufacturer’s instructions.

### 2.9. Western Blotting

In total, 10 µg of cell extract protein was vortexed and centrifuged at 10,000× *g* for 10 min, boiled in NuPAGE^®^ LDS sample buffer with a reducing agent for 5 min at 95 °C and loaded onto a 10% Bolt™ Gel (Invitrogen^TM^, Thermo Fisher Scientific Australia Pty Ltd., VIC, Australia). The gel was electrophoresed for 45 min at a constant 130 V (400 mA) and transferred to a polyvinylidene fluoride (PVDF) membrane using an iBlot Transfer Stack and iBlot Western transfer system (Invitrogen^TM^, Thermo Fisher Scientific Australia Pty Ltd., VIC, Australia) as per the manufacturer’s instructions. The transfer membrane was left to air dry and then rehydrated with methanol and rinsed in distilled water. Total protein was detected by incubating the membranes in REVERT Total Protein Stain (LiCor, NE, USA) for 5 min at RT, with gentle rocking. The membranes were then washed twice with REVERT Total Protein Wash Solution (LiCor, NE, USA), rinsed in distilled water, and immediately imaged on the Odyssey Clx (LiCor, NE, USA). The membranes were then incubated in REVERT Total Protein Stain Reversal (926-11010, LiCor, NE, USA) for 5 min at RT with gentle rocking, washed in distilled water, and blocked by incubation in either 3% BSA TBS-T or Odyssey Blocking Buffer (LiCor, NE, USA) for 1 h at RT. Primary antibodies were incubated overnight at 4 °C in sealed plastic and gently rotated. The membranes were washed three times in TBS-T for 4 min at RT. The secondary antibody (IRDye 680RD or IRDye 800CW) was incubated in the dark for 1 h at RT with gentle rocking. The membranes were rinsed in distilled water and imaged on an Odyssey Clx (LiCor, NE, USA). All protein expression was normalised to total protein.

### 2.10. siRNA Knockdown

SMARTpool ON-TARGETplus siRNA was purchased from DharmaCon Inc. (GE Lifesciences, NSW, Australia) EZR (L-017370-00-0005); RDX (L-011762-00-0005); MSN (L-011732-00-0005); Non-targeting Pool (DHA-D-001810-10-05); GAPDH Control Pool (DHA-D-001830-10-05)). Sterile coverslips were inserted into a 6-well plate. The cells were seeded at approximately 1 × 10^5^ cells/cm^2^ and reverse transfected using Lipofectamine^®^ RNAiMax (Invitrogen^TM^, Thermo Fisher Scientific Australia Pty Ltd., VIC, Australia) as per the manufacturer’s instructions with 25 nM siRNA for 48 h. The cells were washed in ice-cold PBS, and the coverslips were removed and prepped for scanning electron microscopy as above. The remaining cells were prepped for protein extraction as above. 

### 2.11. Cell Viability Assay

Cell culture media was replenished at 1/10 volume of Resazurin (BioReagent, CAS 62758-13-8, Sigma-Aldrich Pty Ltd., NSW, Australia), and the cells were then incubated at 37 °C for 2–4 h. The plate was subsequently read on an Enspire Plate reader (Perkin Elmer, Australia).

### 2.12. Statistical Analysis

Visual representation and data analysis were performed using GraphPad Prism 10 (version 10.01.00). Kruskal–Wallis testing was performed.

## 3. Results

### 3.1. Prostate Cancer Cells Communicate via Tunnelling Nanotubes and Extracellular Vesicles

**Figure 1:**
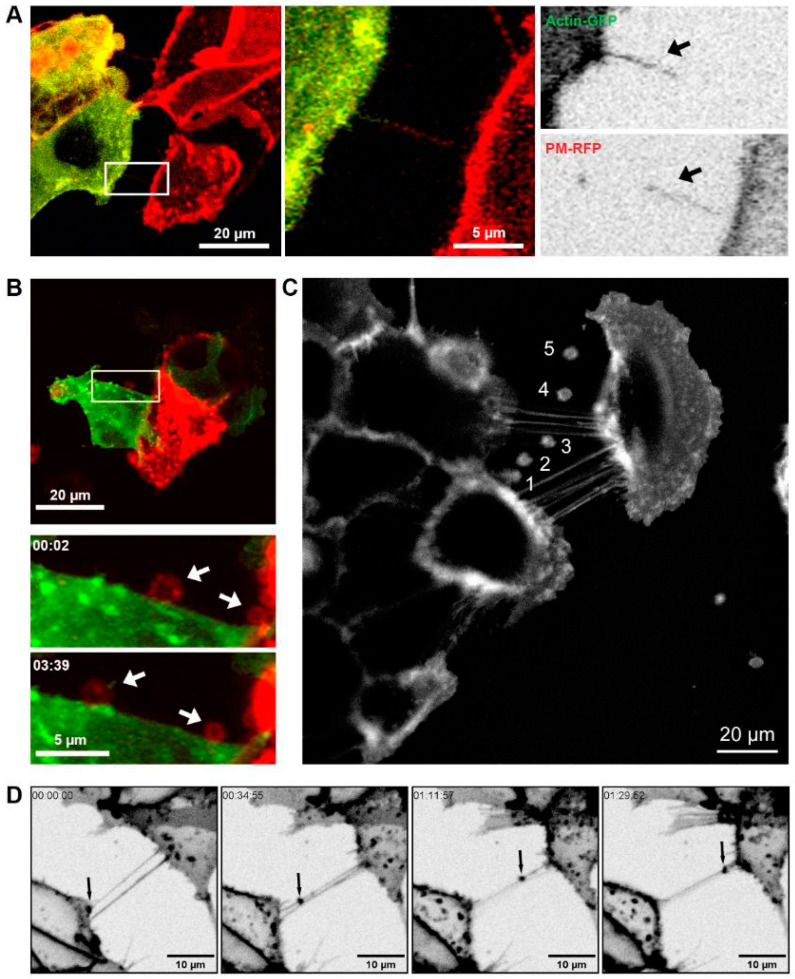
TNTs and EVs mediate intercellular communication. (**A**) Representative live cell images of co-cultured 22Rv1 prostate cancer cells differentially labelled with either actin (green) or CellMask™ Plasma Membrane (PM) stain (red). Merged and individual channel images of a highlighted region of interest are shown at one time frame. Arrows indicate the respective contributions to the tunnelling nanotube. (**B**) Representative live cell images of differentially labelled 22Rv1 cells expressing Lamp1-RFP and actin-GFP co-cultured together. Individual frames at different time points of a highlighted region of interest are shown. Arrows indicate the movement of the Lamp1-RFP positive vesicles. (**C**) Pictorial representation of vesicle budding and staged release (1–5) from a DU145 cell stained with CellMask™ PM dye. Image created from the overlay and merge of area of interest from frames captured at time points 1; 8 s, 2; 96 s, 3; 161 s, 4; 249 s, 5; 379 s. (**D**) Live cell images showing individual frames at different time points of 22Rv1 prostate cancer cells labelled with CellMask™ PM stain. Arrows indicate the movement of a CellMask™ positive vesicle in a tunnelling nanotube.

To investigate TNTs and EVs as modes of intercellular communication between prostate cancer cells, 22Rv1 or DU145 cells were labelled with different dyes or transfected with fluorescently tagged proteins to visualise cellular constituents using confocal microscopy and live cell imaging. TNTs were observed as actin-positive protrusions from the plasma membrane (Figure 1B). 22Rv1 cells transfected with CellLight BacMam actin-GFP were co-cultured with differentially labelled 22Rv1 cells either transfected with CellLight BacMam PM-RFP or Lamp1-RFP DNA plasmid. TNTs were observed protruding from and connecting with the differentially labelled sub-populations of cells, and the subsequent TNT conduits contained fluorescent signals from both actin-GFP and PM-RFP (Figure 1A and Video S1). Lamp1-RFP-positive EVs were observed rolling along the external face of the plasma membrane of adjacent actin-GFP-positive 22Rv1 cells (Figure 1B and Video S2). Cellular content transfer was observed between differentially labelled cells as indicated by co-fluorescence (yellow; Figure 1A,B). Vesicular budding/release and TNT interactions were observed in DU145 cells stained with CellMask PM dye (Figure 1C and Video S3). The movement of a vesicle along a TNT was visualised using a CellMask™ membrane stain and involved the coordinated transit of two TNTs and EVs between the two adjacent 22Rv1 prostate cancer cells (Figure 1D).

### 3.2. Communication Between Prostate Cancer Cells and Non-Malignant Cells or Macrophages

**Figure 2:**
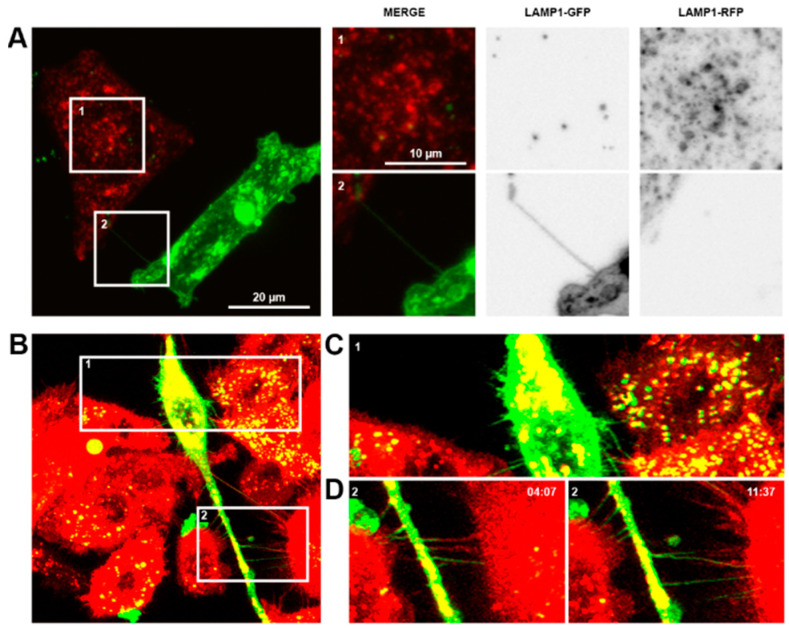
Communication between prostate cancer cells and non-malignant cells or macrophages. (**A**) Representative confocal images of 22Rv1 prostate cancer cells labelled with Lamp1-GFP (green) co-cultured with PNT1a non-malignant cells labelled with Lamp1-RFP (red). Merged and individual channel images of two highlighted regions of interest shown. (**B**) Representative live cell micrographs of THP-1 macrophages labelled with DiO (green) co-cultured with PC-3 prostate cancer cells labelled with DiD (red). Individual frames at different time points of two highlighted regions of interest are shown (**C**,**D**).

22Rv1 prostate cancer cells and PNT1a non-malignant prostate cells transiently expressing, respectively, Lamp1-GFP and -RFP were co-cultured and imaged (Figure 2A). GFP-positive TNTs were observed originating from prostate cancer cells and initiating contact with non-malignant PNT1a cells. Intracellular transfer of material from prostate cancer cells to PNT1a cells was observed by yellow co-fluorescence (Figure 2(A1)). This exchange was also observed between LNCaP and PNT1a cells (Figure S2). Tunnelling nanotubes/cell bridges were also formed between co-cultured PC-3 prostate cancer cells labelled with lipophilic DiD (red) dye and THP-1 macrophages labelled with DiO (green) (Figure 2B). There was a significant bidirectional exchange of lipophillic labels between PC-3 cells and THP-1 macrophages as evident by the yellow fluorescence in TNTs, EVs, and each cell type (Figure 2C,D). In live cell images, co-fluorescence was observed within the EVs and showed movement of the internal contents (Video S4). TNTs/cell bridges between the cell types were observed connecting and breaking over time frames from live imaging capture (Figure 2B and Video S4). Of particular note, structures resembling cell bridges (shorter and wider connections) were observed to extend into longer and thinner connections that resembled TNTs (Figure 2B and Video S4).

### 3.3. Prostate Cancer Cells Transfer Intracellular Contents and Organelles via TNTs/Cellular Bridges

**Figure 3:**
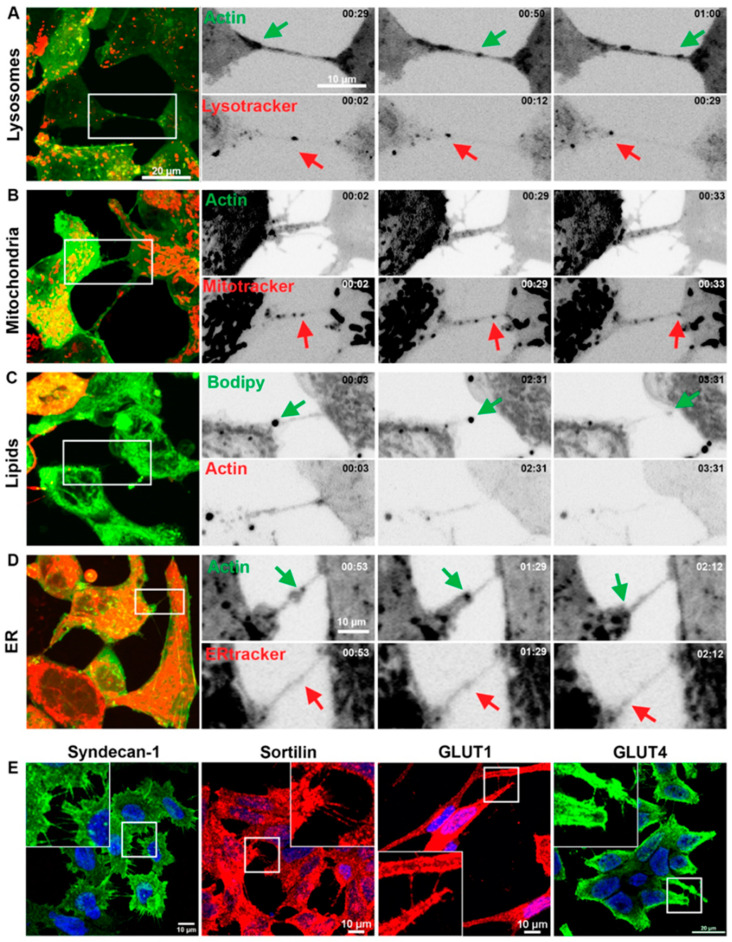
Vesicular compartments/organelles and protein transfer between prostate cancer cells via TNTs/cellular bridges. Representative images showing 22Rv1 cells expressing fluorescently tagged F-actin and stained with LysoTracker red (**A**), Mitotracker red (**B**), BODIPY (**C**), and ER tracker red (**D**). The highlighted region of interest (ROI) shows individual frames cropped from areas at different time points. (**E**) Representative confocal images of prostate cancer cell lines showing immunolabelling of Syndecan-1, Sortilin, GLUT1, and GLUT4 detected in TNTs/cellular bridges. Arrows highlight the movement of the respective vesicles.

To characterise TNT/cellular bridge cargo transport between cells, 22Rv1 cells were labelled with F-actin cytoskeletal stain and either Lysotracker lysosomal stain (Figure 3A and Video S5), Mitotracker mitochondrial stain (Figure 3B and Video S6), ER tracker endoplasmic recticulum (ER) stain (Figure 3C and Video S7), or BODIPY-stained lipid droplets (Figure 3D and Video S8). Using live cell imaging, the transport of this organelle cargo to neighbouring cells was observed via F-actin positive TNT/cellular bridges (Figure 3A–D). Importantly, the bidirectional transport of either Lysotracker or actin-stained vesicles (Lysotracker red in one direction and green actin in the opposite direction) within a TNT/cell bridge was observed between 22Rv1 cells (Figure 3A). Mitochondria were also observed undergoing bidirectional traffic between cells. Using immunofluorescent labelling, the membrane-associated proteins Syndecan-1 and Sortilin, plus the glucose transporters GLUT1 and GLUT4, were observed inside/associated with TNT/cellular bridge structures connecting prostate cancer cells (in PC-3, LNCaP, LNCaP, and 22Rv1 cells, respectively) (Figure 3E).

### 3.4. R1881 Treatment Stimulated Changes in the Cell Surface Morphology of LNCaP Prostate Cancer Cells

**Figure 4:**
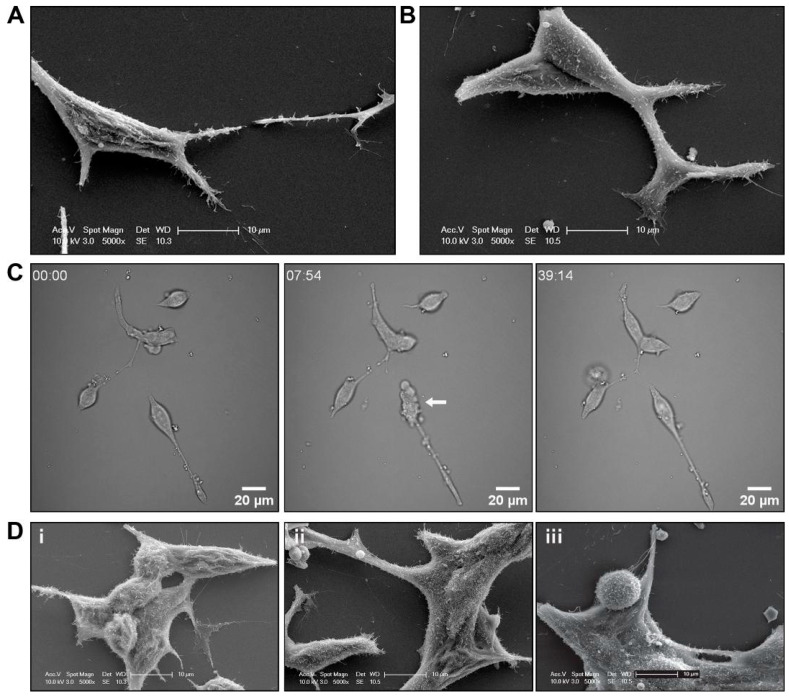
R1881 treatment induced cell surface morphology changes to LNCaP prostate cancer cells. Representative scanning electron microscopy (SEM) micrographs of LNCaP prostate cancer cells treated with (**A**) vehicle or (**B**) 10 nM R1881. (**C**) Brightfield live cell images of LNCaP cells immediately after treatment with R1881. Representative frames at individual time points shown. (**D**) Representative SEM micrographs of LNCaP prostate cancer cells treated with (**i**) vehicle, (**ii**) 10 nM R1881 for 5 min, or (**iii**) for 20 min.

Given the importance of the AR in normal cell growth and its critical role in the progression of prostate cancer, we investigated potential cell morphology changes induced by treatment with the synthetic androgen R1881 on androgen-sensitive LNCaP cells. In androgen-deprived cells, SEM revealed structures resembling membrane ruffles on LNCaP cells (Figure 4A,D(i)), which changed after the addition of R1881, resulting in a smoother membrane surface topography (Figure 4B,D(ii,iii)). In response to R1881 treatment, immediate changes in membrane morphology, including membrane blebbing, were observed, which resolved after approximately 8 min post-treatment (Figure 4). Interestingly, the morphological changes could be visualised by SEM during a fixed time chase, whereby extracellular vesicles docked to the cell surface appeared to be increased, and membrane ruffling again appeared to be reduced after R1881 treatment (Figure 4D). 

### 3.5. Androgen Receptor Localisation and Transport

**Figure 5:**
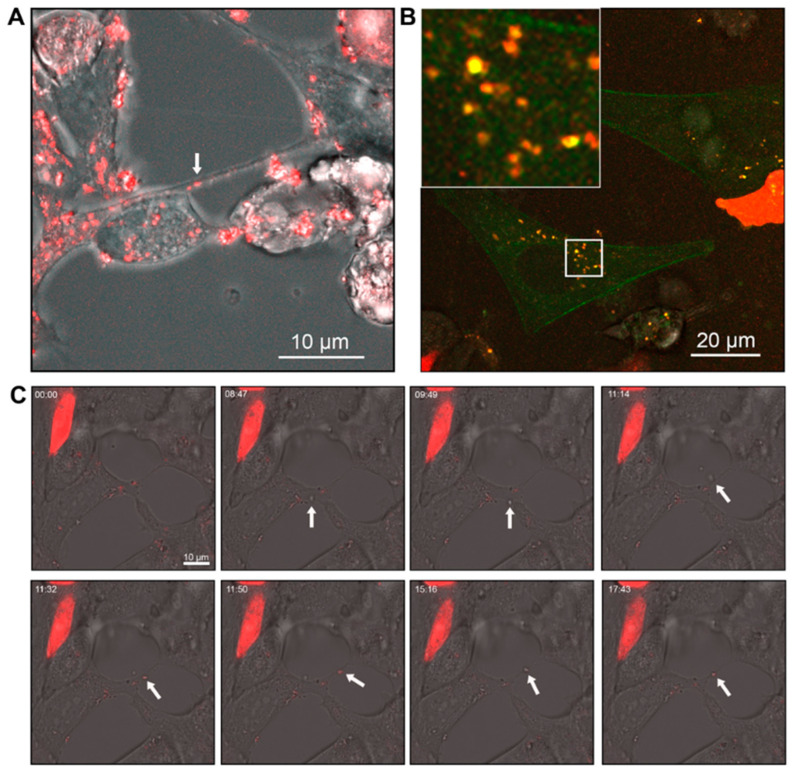
Androgen receptor (AR) co-localises within vesicular structures for intra- and intercellular transport. (**A**) Merged brightfield and AR-mCherry fluorescence showing AR in vesicles within LNCaP cells and within TNTs/cellular bridges. Arrow highlights AR-mCherry positive vesicles. (**B**) Representative confocal images of PNT1a cells transfected with actin-GFP and 22Rv1 cells transfected with AR-mCherry. (**C**) Brightfield live cell images of LNCaP cells transfected with AR-mCherry immediately after treatment with R1881. Representative frames at individual time points shown. Arrows highlight movement of an AR-mCherry positive vesicle.

Following our observation that R1881 treatment induced changes in membrane and vesicular topography, we transfected LNCaP cells with a uniquely designed AR plasmid (Figure S1) to investigate the receptor transport and its localisation. The presence of the AR-mCherry protein at the correct size was confirmed post-transfection via Western blot, with AR-negative BxPC-3 cells as a comparison (Figure S3). AR-mCherry was observed in association with vesicular structures through out LNCaP cells and was transported between cells in vesicles transiting along TNTs/cellular bridges (Figure 5A). Vesicular-associated AR-mCherry was also observed within 22Rv1 prostate cancer cells (Figure S4A). Co-culture experiments between non-malignant PNT1a cells transfected with actin-GFP, and 22Rv1 cells transfected with AR-mCherry (Figure 5B), revealed that AR-mCherry was transferred to neighbouring cells. AR transport was also observed between LNCaP and PNT1a cells (Figure S4B). Extracellular docking of AR-mCherry-positive vesicles onto LNCaP cells was observed (Figure 5C).

### 3.6. Ezrin Phosphorylation and Cellular Localisation Is Altered Following R1881 Treatment

**Figure 6:**
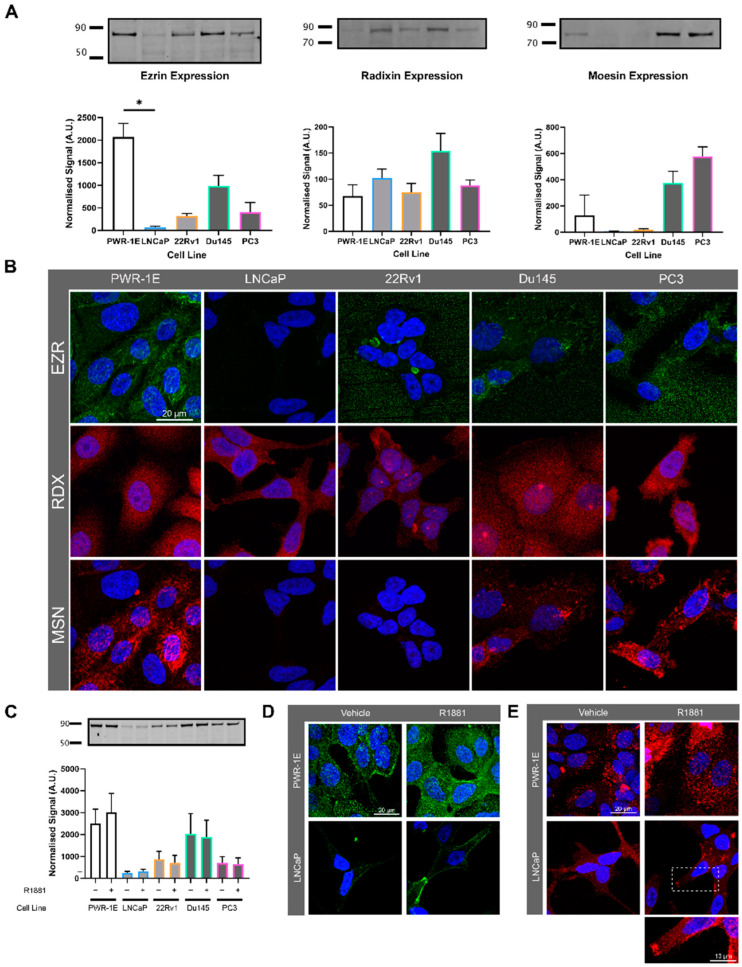
Prostate cell line ERM protein expression and localisation. (**A**) Endogenous expression of ezrin, radixin, and moesin (ERM) in prostate cell lines detected by Western blotting. The signal was quantified by normalising to total protein. One-way ANOVA performed with Kruskal-Wallis Test * *p* < 0.05 (**B**) Representative confocal microscopy images characterising ERM expression and localisation in prostate cell lines. (**C**) Western blot of prostate cell lines showing expression of ERM with either vehicle (0.01% *v*/*v* EtOH) or 10nm R1881 treatment for 48 h. Signal quantified by normalising to total protein. (**D**) Representative confocal images of PWR-1E and LNCaP cells treated with 10nM R1881 or vehicle and labelled with pan-EZR antibody. (**E**) Representative confocal images of PWR-1E and LNCaP cells treated with R1881 or vehicle and labelled with phosphorylated-EZR antibody. Original images of can be found in Supplementary Materials.

Ezrin is involved in plasma membrane and actin crosslinking, and is localised at the site of tunnelling nanotube initiation, prompting us to investigate ezrin and it’s functionally redundant protein family members moesin and radixin, and their role in modulating cell surface morphology in prostate cancer cells. Ezrin and moesin expression was reduced in androgen-sensitive LNCaP and 22Rv1 prostate cancer cell lines compared to androgen-sensitive non-malignant PWR-1E prostate cells (Figure 6A,B). Ezrin is activated and phosphorylated in the presence of R1881 [23]. Although R1881 treatment did not significantly effect ezrin protein expression (Western blot; Figure 6C), immunofluorescence showed that the cellular localisation of ezrin protein was altered in PWR-1E and LNCaP cells with R1881 treatment, increasing its membrane localisation (Figure 6D,E). Moesin and radixin expression did not change with R1881 treatment (Figure S5).

### 3.7. Ezrin Knockdown Alters Prostate Cancer Cell Surface Morphology

**Figure 7:**
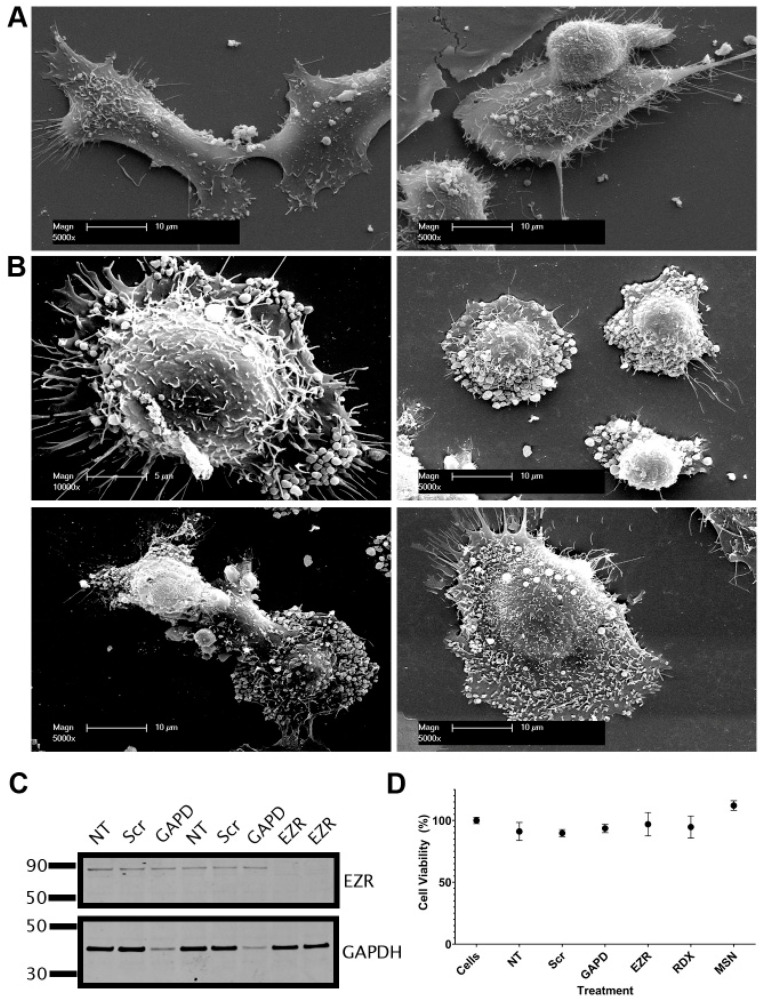
Knockdown of ezrin alters cell surface morphology but does not induce cell death. Representative scanning electron microscopy (SEM) images of DU145 cells with (**A**) control scramble siRNA and (**B**) ezrin siRNA knockdown. (**C**) Western blot of DU145 cell lysates following siRNA knockdown. (**D**) Cell viability values following siRNA knockdown. NT; no transfection, Scr; control scramble siRNA, GAPD; glyceraldehyde-3-phosphate dehydrogenase (GAPDH) control siRNA, EZR; ezrin, RDX; radixin, MSN; moesin. Original images of can be found in Supplementary Materials.

Using androgen-insensitive DU145 prostate cancer cells, which have higher endogenous ezrin expression (Figure 6A), we knocked down ezrin and observed cells under SEM to investigate the effect on TNT and EV morphology. Ezrin knockdown in DU145 cells induced a significant alteration in surface morphology, with an increased appearance of vesicular structures on the plasma membrane (Figure 7B) compared to control untreated DU145 cells (Figure 7A), which was confirmed via Western blot analysis (Figure 7C). The cells appeared to adopt an apoptotic-like phenotype with vesicular protrusions evident on the cells, but the cell viability was not altered (Figure 7D).

## 4. Discussion

While EVs and TNTs/cellular bridges are well-recognised modes of intercellular communication [24,25], there has been limited investigation of these conduits for cellular resources and information transfer in the context of prostate cancer pathogenesis. We provide evidence that TNTs/cellular bridges and EVs are commonly observed in prostate cancer cells and may facilitate intercellular communication. Here, we provide evidence of dynamic, real-time interactions in which prostate cancer cells appear to use to communicate with surrounding cancer cells and to modulate and exploit other cells that reside within their microenvironment [26].

Prostate cancer cells establish and utilise a dynamic network of cargo exchange that contributes to intercellular communication, resource sharing/sequestering, and metabolic reprogramming. TNTs/cellular bridges and EVs were observed transferring cellular contents, critical organelles, and other protein cargo involved in, for example, biosynthesis, intracellular transport, signalling, energy sensing/storage, and degradation. Notably, these interactions involved the bidirectional transfer of various organelles and cargo within singular TNTs/cellular bridges, and included mitochondrial and endosomal–lysosomal compartments. While TNTs and EVs are thought to be independent modes of intercellular communication, EVs were observed interacting with and moving along the external side of TNTs/cellular bridges. This suggests that EVs can be captured by intercellular connections and may direct cargo to specific sites of interaction. Indeed, it is not currently known whether EVs have to use specific docking sites to facilitate exchange. Interestingly, we also observed prostate cancer cells interacting with other cell types, indicating that the information and resource exchange network is not just restricted to interactions between cancer cells. Considerable work is required to establish the functional consequences of these transmission networks, but metabolic programming and the transfer of primary resources to drive cancer progression would appear highly likely [27].

Dynamic formation and breakage of TNTs was observed between cells, which has been previously postulated to involve donating and accepting membrane proteins and lipids [28]. We also observed short cellular bridge structures extending into longer connections that ultimately resembled TNTs. In addition, long TNT structures were observed probing the surrounding environment or interacting with other cells to form stable connections. While some reports have referred to TNTs as a cell culture phenomenon, we have observed TNTs between circulating tumour cells (CTCs) and also involving immune cells (Ward et al., publication in preparation). Here, we demonstrated that prostate cancer TNTs/cellular bridges contain critical integral membrane proteins, presumably by providing continuity between the plasma membrane of different cells and enabling the internal and external transfer of membrane and protein constituents. Sortilin, a sorting receptor which controls trans-Golgi transport into the vesicular compartments within cell networks [29], and Syndecan-1, a transmembrane proteoglycan involved in cell proliferation, migration, and cell–matrix interactions [30,31], were both present in TNTs. These biomarkers have recently been implicated in prostate cancer pathogenesis and have been established as components of primary pathogenesis, which can be used to facilitate diagnosis and prognosis [32,33]. Prostate cancer cells therefore establish a communication and exchange network that is integrally linked to the pathogenic process. 

Metabolic reprogramming is crucial to sustaining the energy demands of prostate cancer development and progression, which is equally important for adapting to a changing microenvironment (e.g., during migration or the metastatic cascade) [34]. In normal prostate growth, zinc accumulation inhibits mitochondrial aconitase to limit Krebs cycle metabolism, and the main energy source for ATP production is provided by glycolysis [35]. However, prostate cancer cells have been shown to adapt to rapidly changing microenvironment conditions, and instead utilise fatty acids produced by lipogenesis to produce cellular energy. Mitochondrial energy production is exploited by prostate cancer cells at different stages of growth/progression (e.g., to produce ATP and generate excess ROS during early cancer development), but prostate cancer cells also have a propensity for glycolysis, even in the presence of oxygen (Warburg effect) [35,36,37]. This critical aspect of metabolic programming is fundamental in prostate cancer biology and likely involves adapting to different environmental conditions, such as hypoxia, by changing metabolism to enable progression and survival. Here, we showed the capacity for prostate cancer cells to transfer organelles that are involved in energy sensing and production including mitochondria, lipid droplets, and endosomes/lysosomes, which were transported via TNTs/cellular bridges and EVs. The glucose transport receptors, GLUT1 and GLUT4, were also observed within TNTs and EVs, which also has implications for glucose uptake and cellular metabolism. TNTs/cellular bridges and EVs are likely to support dynamic changes in energy metabolism required for rapid cellular division, adapting to hypoxic conditions, and for transitioning between different environments during cancer cell metastasis.

We have previously demonstrated altered endosome–lysosome biogenesis and expression of vesicular trafficking machinery in prostate cancer [22,38]. With this evidence, and, here, the import and export of mitochondria through TNTs and cellular bridges, it is not unreasonable to postulate that prostate cancer cells can also transfer mitochondria in EVs. The delivery of mitochondria, which may also be pre-programmed, would enable recipient cells to acquire an advantageous energy supply or to adapt surrounding cells to a specific metabolic phenotype. Mitochondrial transfer via TNTs has been implicated in increasing aerobic respiration in recipient cells supporting this concept [39]. Changing the expression of glucose transporters and other metabolic machinery could also augment these metabolic profiles, which may promote accelerated progression through the cell cycle, providing an opportunity to grow exponentially and potentially introduce further mutations in cells. This concept not only applies to neighbouring prostate cancer cells, the immediate tumour microenvironment, but importantly includes immune cells that rely on specific metabolic control. The TNT/cellular bridge modification of the local microenvironment and EV access to stromal cells, blood vessels, and distal tissue is likely to be critical during metastasis and to generate a premetastatic niche. Combining metabolic signatures and programming with the transfer of ER, lipid droplets, endosomes, and lysosomes is likely to enable specific changes in biosynthesis, provide lipids for membrane and hormone production, transfer aberrant signalling, and change degradative potential in target cells.

The biology of the AR has been integrally linked to metabolic reprogramming and transcriptional changes in prostate cancer, and here we show an association of this critical receptor with endosomes–lysosomes and its intra- and intercellular transport. Androgen receptor activity is in part responsible for maintaining prostate metabolism, and therefore any alterations to the receptor, including splice variants, can have significant implications to the metabolic profile of prostate cancer cells. We have previously shown that androgen treatment can impact on glucose uptake in LNCaP prostate cancer cells by inducing an upregulation of sortilin and GLUT1 [32]. There are other organelles and proteins that can also actively contribute to the metabolic activity of the prostate epithelium, including mitochondria, endoplasmic reticulum, syndecan, sortillin, and glucose transport proteins such as GLUT4. The role of AR in controlling the transcription and translation of critical metabolic and trafficking machinery is important, and therefore its transfer between cancer cells and other cells adds another dimension to this critical cell biology.

The significance of AR in prostate cancer biology has been well documented and, as a result, multiple therapies target this important pathway. Under normal physiological conditions, full-length AR is thought to reside within the cytoplasm prior to its activation; however, our study indicates that a significant amount of AR may reside in association with endosome–lysosome vesicles within the cell. AR overexpression plasmids [40] have consistently lacked the canonical full-length human AR mRNA that contains a considerable 3′ UTR that controls RNA stability, translation, cellular trafficking, and localization [41]. As this may have significant implications when investigating cellular localisation and transport, we developed a new plasmid that contained all of the appropriate regulatory regions of the AR mRNA. AR signalling relies on its transfer to the nucleus, and whilst important in biological processes of both normal and cancerous conditions, the mechanistic process of AR trafficking within cells is yet to be determined. Our study shows a mode of transport both prior to and immediately after R1881 treatment, which may provide an alternate target for therapies. Androgens can activate both a rapid and late response pathway in the AR signalling cascade, with the former dependent on the Raf-1-MEK pathway [42], resulting in the upregulation of pro-survival, proliferation, and metastatic gene expression, pathways that contribute to key hallmarks of cancer. The transfer of the AR to neighbouring cells and to distal sites may therefore modulate the surrounding and distant microenvironment to promote the metastatic cascade. 

ERM proteins have the capacity to interact with and connect the plasma membrane to filamentous actin [43], and actin-like filaments are found in TNTs [44] and EVs [45,46]. Ezrin is an androgen-regulated gene [23] and has been suggested as a target for cancer diagnosis and therapy due to its involvement in cancer progression, metastasis, and patient survival cancers [47]. This prompted us to investigate the involvement of ERM proteins in TNT and EV formation. In contrast to previous reports, we showed that ezrin had reduced expression in prostate cancer cells compared to non-malignant cells, albeit with variable expression between different prostate cancer cell lines. Importantly, androgen treatment stimulated the localisation of ezrin to the plasma membrane, whereby it may be fulfilling its role to link actin and the plasma membrane. The knockdown of ezrin had a profound effect on DU145 prostate cancer cells, inducing morphological changes and the appearance of EV-like structures at the cell surface. There were also marked differences in moesin expression in androgen sensitive (low) to androgen-independent cell lines (high), indicating a response to AR activity. The dynamic balance between ERM proteins and links with the cytoskeleton may be a critical component for regulating EV and TNT formation, thereby controlling either membrane protrusions or vesicular formation at the cell surface.

Cellular communication is critical to normal cellular function, and TNTs have been well described in the recent literature, particularly in stress-induced situations, including inflammatory conditions which support their function in pathophysiological conditions. The transferred cargo is vast, with the direct implications in disease not yet fully understood; however, the organelles and proteins we have observed in this study suggest a role in energy metabolism and resource sharing. It is evident that there is a requirement for molecular machinery, including filament extension and vesicular trafficking machinery, to facilitate the transfer process, which also aids in TNT identification. Given the varying types of cargo that were observed, we suggest that TNTs can transfer all cellular components, with transportation being fuelled by ATP generated from glucose or lipid metabolism or any other metabolic activity in the cell of origin. TNTs remain a novel form of cellular communication and still require further studies to investigate the direct implications of categorised cargo transfer to enlighten functions in disease settings. As indicated by our study, TNTs are capable of transferring various cellular components which have been attributed to PCa pathogeneis. It is therefore critical to further elucidate the molecular mechanisms of TNT formation as well as determining a molecular switch to EV communication in order to identify novel biomarkers or therapeutic targets for PCa patients.

## 5. Conclusions

The direct connection of the cytoplasm for two cells is a fundamental system for cellular cross talk and the exchange of contents, which forms part of an intricate network of communication. The exchange of messages in a paracrine fashion is also key to information and cellular content transfer and involves EVs that can facilitate longer-range transfer. This transport and communication system enables the exchange of organelles, cytokines, chemokines, growth factors, nutrients, raw materials, energy, cytoskeletal structural elements, and signalling messages. We show that prostate cancer cells transport an extensive array of organelle cargo and protein/membrane constituents between cancer cells as well as other cell types. The dynamics of this process when visualised by real-time live cell imaging reveals a complex interplay and mixing of cellular contents. Paramount in this exchange is our unique visualisation of AR intercellular traffic, which has profound significance due to the known consequences of androgen biology in cancer progression and response to treatment. The mechanisms regulating TNT and EV biology and the transport of cargo are yet to be fully elucidated, but clearly the link between cytoskeletal protein machinery and cell membranes is an important focus for future investigation.

## Figures and Tables

**Table 1:** Commercial antibody and imaging reagents. RT; room temperature, OBB; Odyssey Blocking Buffer.

Reagent	Catalogue Number	Company	Stain	IF	Western Blot
Cellmask™ Plasma Membrane-RFP Stain	C10046	Invitrogen™ Thermo Fisher Scientific Australia Pty Ltd., VIC, Australia	1:1000, 30 m, 37 °C		
CellLight™ Plasma Membrane-RFP BacMam 2.0	C10608	Invitrogen™ Thermo Fisher Scientific Australia Pty Ltd., VIC, Australia			
CellLight™ Actin-GFP BacMam 2.0	C10582	Invitrogen™ Thermo Fisher Scientific Australia Pty Ltd., VIC, Australia			
Vybrant™ DiO Cell-Labeling Solution	V22886	Invitrogen™ Thermo Fisher Scientific Australia Pty Ltd., VIC, Australia	1:200, 20 m, 37 °C		
Vybrant™ DiD Cell-Labeling Solution	V22887	Invitrogen™ Thermo Fisher Scientific Australia Pty Ltd., VIC, Australia	1:200, 20 m, 37 °C		
LysoTracker Red DND-99	L7528	Invitrogen™ Thermo Fisher Scientific Australia Pty Ltd., VIC, Australia	1:1000, 30 m, 37 °C		
MitoTracker^®^ Red CMXRos	M7512	Invitrogen™ Thermo Fisher Scientific Australia Pty Ltd., VIC, Australia	1:1000, 30 m, 37 °C		
ER-Tracker™ Red	E34250	Invitrogen™ Thermo Fisher Scientific Australia Pty Ltd., VIC, Australia	1:1000, 30 m, 37 °C		
BODIPY^®^ 493/503	D3922	Invitrogen™ Thermo Fisher Scientific Australia Pty Ltd., VIC, Australia	1:1000, 30 m, 37 °C		
Anti-Ezrin mouse antibody	ab4069	abcam, Cambridge, UK		1:500 overnight, 4 °C	1:500, overnight, 4 °C OBB
Anti-Radixin rabbit antibody	2636S	Cell Signalling/NEB Australia		1:500 overnight, 4 °C	1:500, overnight, 4 °C 3% BSA
Anti-Moesin rabbit antibody	3150S	Cell Signalling/NEB Australia		1:500 overnight, 4 °C	1:500, overnight, 4 °C 3% BSA
Anti-pEzrin rabbit antibody	3726S	Cell Signalling/NEB Australia		1:1000, overnight, 4 °C	
Anti-AR rabbit antibody	ab108341	abcam, Cambridge, UK		1:1000, overnight, 4 °C	1:1000, overnight, 4 °C 3% BSA
Anti-GAPDH mouse HRP conjugated antibody	G9295	Merck, Sigma-Aldrich Pty Ltd., NSW, Australia			1:10,000, 1 h, RT
Anti-Sortilin mouse antibody	ab16640	abcam, Cambridge, UK		1:1000, overnight, 4 °C	
Anti-Syndecan-1 mouse antibody	ab34164	abcam, Cambridge, UK		1:250, overnight, 4 °C	
Anti-GLUT1 mouse antibody	ab40084	abcam, Cambridge, UK		1:100, overnight, 4 °C	
Anti-GLUT4 mouse antibody	ab35826	abcam, Cambridge, UK		1:250, overnight, 4 °C	
Anti-rabbit AlexaFluor 647 conjugated secondary antibody	a31573	Invitrogen™ Thermo Fisher Scientific Australia Pty Ltd., VIC, Australia		1:1000, 1 h, RT	
Anti-mouse AlexaFluor 488 conjugated secondary antibody	a21202	Invitrogen™ Thermo Fisher Scientific Australia Pty Ltd., VIC, Australia		1:1000, 1 h, RT	
Hoechst	33342	Invitrogen™ Thermo Fisher Scientific Australia Pty Ltd., VIC, Australia			
Rabbit IRDye 800CW IgG secondary antibody	926-32211	LiCor, NE, USA			1:10,000, 1 h, RT
Mouse IRDye 680RD IgG secondary antibody	926-68070	LiCor, NE, USA			1:10,000, 1 h, RT

## Data Availability

The original contributions presented in this study are included in the article/Supplementary Materials. Further inquiries can be directed to the corresponding author(s).

## Supplementary Materials

The following supporting information can be downloaded at https://www.mdpi.com/article/10.3390/biom15010023/s1. Figure S1. Annotated plasmid DNA gene map for pcDNA3.1_AR-mCherry. Figure S2. Representative confocal images of LNCaP prostate cancer cells labelled with Lamp1-GFP (green) co-cultured with PNT1a non-malignant cells labelled with Lamp1-RFP (red). Figure S3. Confirmation of AR expression plasmid construct via Western blot of AR in AR-negative BXPC-3 pancreatic cell lines, and 22Rv1 prostate cancer cells either non-transfected or transfected with AR-mCherry. Figure S4. Representative live cell images of (A) AR-mCherry associated with extracellular vesicles in 22Rv1 cells and (B) LNCaP cells expressing AR-mCherry co-cultured with PNT1a cells transfected with actin-GFP. Figure S5. RDX and MSN protein expression are not affected by R1881 treatment. Endogenous Radixin and Moesin protein were detected by Western blot post R1881 treatment and corresponding signal quantified by normalising to total protein stain. Video S1. Live cell imaging video of co-cultured 22Rv1 prostate cancer cells differentially labelled with either actin (green) or CellMask™ Plasma Membrane (PM) stain (red). Video S2. Live cell imaging video of differentially labelled 22Rv1 cells expressing Lamp1-RFP and actin-GFP co-cultured together. Video S3. Live cell imaging video of DU145 cells stained with CellMask™ PM dye showing vesicle budding and TNT connection. Video S4. Live cell imaging video of THP-1 macrophages labelled with DiO (green) co-cultured with PC-3 prostate cancer cells labelled with DiD (red). Video S5. Live cell imaging video showing 22Rv1 cells expressing fluorescently tagged F-actin and stained with LysoTracker red. Video S6. Live cell imaging video showing 22Rv1 cells expressing fluorescently tagged F-actin and stained with Mitotracker red. Video S7. Live cell imaging video showing 22Rv1 cells expressing fluorescently tagged F-actin and stained with ER tracker red. Video S8. Live cell imaging video showing 22Rv1 cells expressing fluorescently tagged F-actin and stained with BODIPY.
