# Supplementary material for: Extracellular Vesicles and Tunnelling Nanotubes as Mediators of Prostate Cancer Intercellular Communication
Source: Biomolecules. 2024 Dec 27;15(1):23. doi: 10.3390/biom15010023 (PMC11762852; doi:10.3390/biom15010023)
Supplement: Supplementary file 1 [file biomolecules-15-00023-s001.zip › biomolecules-3305814-supplementary new/biomolecules-3305814-supplementary.pdf]

biomolecules

Supplementary Materials

Extracellular Vesicles and Tunnelling Nanotubes as Mediators of Prostate Cancer Intercellular Communication

Jessica K. Heatlie [1,*], Joanna Lazniewska [1], Courtney R. Moore [1], Ian R. D. Johnson [1], Bukuru D. Nturubika [1], Ruth Williams [1], Mark P. Ward [2], John J. O'Leary [2], Lisa M. Butler [3,4] and Doug A. Brooks [1,2,*]

1 Clinical and Health Sciences, University of South Australia, Adelaide, SA 5000, Australia; joanna.lazn@gmail.com (J.L.); courtney.moore@unisa.edu.au (C.R.M.); ian.johnson@ext.esa.int (I.R.D.J.); bukuru.nturubika@mymail.unisa.edu.au (B.D.N.); ruth.williams@unisa.edu.au (R.W.)

2 Department of Histopathology, Trinity College Dublin, D02 PN40 Dublin, Ireland; wardm6@tcd.ie (M.P.W.);
solaoire@gmail.com (J.J.O.)

3 South Australian ImmunoGENomics Cancer Institute and Freemasons Centre for Male Health and Wellbeing, University of Adelaide, Adelaide, SA 5005, Australia; lisa.butler@adelaide.edu.au (L.M.B);

4 Solid Tumour Program, Precision Cancer Medicine Theme, South Australian Health and Medical Research Institute, Adelaide, SA 5000, Australia;

* Correspondence: jessica_kate.heatlie@mymail.unisa.edu.au (J.K.H.); doug.brooks@unisa.edu.au (D.A.B.)

Citation: Heatlie, J.K.; Lazniewska, J.; Moore, C.; Johnson, I.R.; Nturubika, B.D.; Williams, R.; Ward, M.P.; O'Leary, J.J.; Butler, L.M.; Brooks, D.A. Extracellular Vesicles and Tunnelling Nanotubes as Mediators of Prostate Cancer Intercellular Communication. *Biomolecules* **2025**, *15*, 23. https://doi.org/10.3390/biom15010023

Academic Editor: Piwen Wang

Received: 25 October 2024
Revised: 4 December 2024 Accepted: 5 December 2024 Published: date

Copyright: © 2024 by the authors. Submitted for possible open access publication under the terms and conditions of the Creative Commons Attribution (CC BY) license (https://creativecommons.org/licenses/by/4.0/).

Figures:

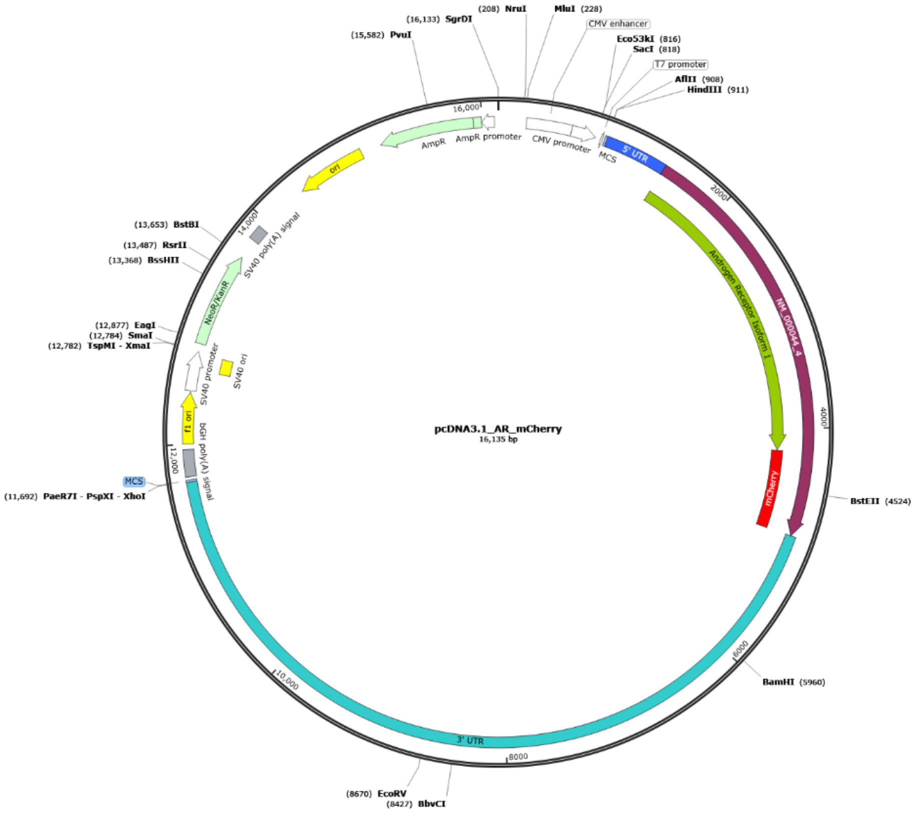

Figure S1. Annotated plasmid DNA gene map for pcDNA3.1_AR-mCherry.

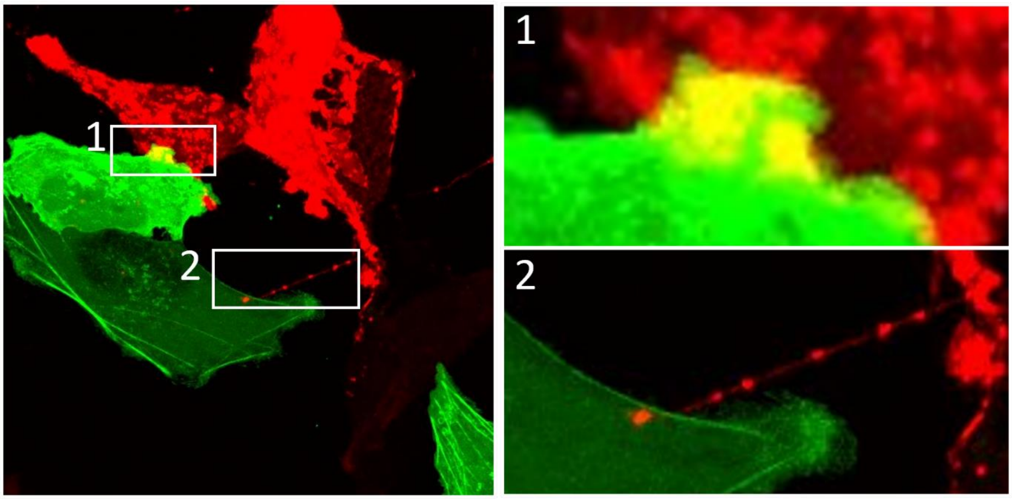

Figure S2. Representative confocal images of LNCaP prostate cancer cells labelled with Lamp1-GFP (green) co-cultured with PNT1a non-malignant cells labelled with Lamp1-RFP (red).

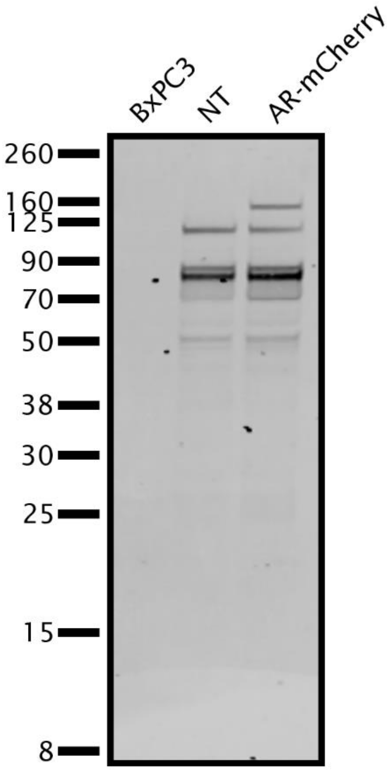

Figure S3. Confirmation of AR expression plasmid construct via western blot of AR in AR negative BXPC-3 pancreatic cell lines, and 22Rv1 prostate cancer cells either non-transfected or transfected with AR-mCherry.

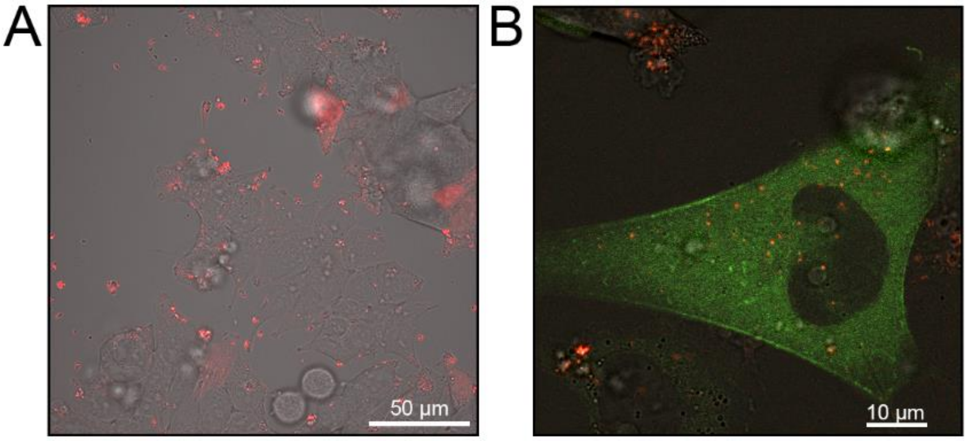

Figure S4. Representative live cell images of (**A**) AR-mCherry associated with extracellular vesicles in 22Rv1 cells and (**B**) LNCaP cells expressing AR-mCherry co-cultured with PNT1a cells transfected with actin-GFP.

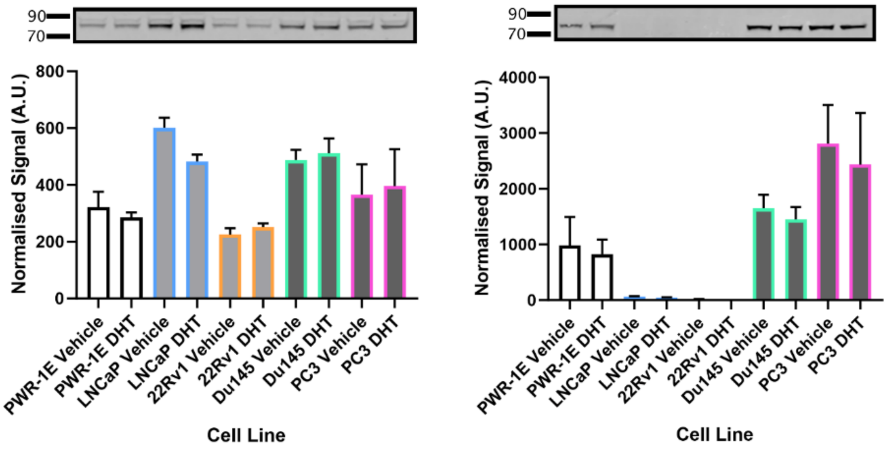

Figure S5. RDX and MSN protein expression are not affected by R1881 treatment. Endogenous Radixin and Moesin protein were detected by Western blot post R1881 treatment and corresponding signal quantified by normalising to total protein stain.

Videos:

Video S1. Live cell imaging video of co-cultured 22Rv1 prostate cancer cells differentially labelled with either actin (green) or CellMask™ Plasma Membrane (PM) stain (red).

Video S2. Live cell imaging video of differentially labelled 22Rv1 cells expressing Lamp1-RFP and actin-GFP co-cultured together.

Video S3. Live cell imaging video of DU145 cells stained with CellMask™ PM dye showing vesicle budding and TNT connection.

Video S4. Live cell imaging video of THP-1 macrophages labelled with DiO (green) co-cultured with PC-3 prostate cancer cells labelled with DiD (red).

Video S5. Live cell imaging video showing 22Rv1 cells expressing fluorescently tagged F-actin and stained with LysoTracker Red.

Video S6. Live cell imaging video showing 22Rv1 cells expressing fluorescently tagged F-actin and stained with Mitotracker Red.

Video S7. Live cell imaging video showing 22Rv1 cells expressing fluorescently tagged F-actin and stained with ER tracker Red.

Video S8. Live cell imaging video showing 22Rv1 cells expressing fluorescently tagged F-actin and stained with BODIPY.
